# Esculetin, a Coumarin Derivative, Prevents Thrombosis: Inhibitory Signaling on PLCγ2–PKC–AKT Activation in Human Platelets

**DOI:** 10.3390/ijms20112731

**Published:** 2019-06-03

**Authors:** Chih-Wei Hsia, Kao-Chang Lin, Tzu-Yin Lee, Chih-Hsuan Hsia, Duen-Suey Chou, Thanasekaran Jayakumar, Marappan Velusamy, Chao-Chien Chang, Joen-Rong Sheu

**Affiliations:** 1 Graduate Institute of Medical Sciences, College of Medicine, Taipei Medical University, Taipei 110, Taiwan; d119106003@tmu.edu.tw (C.-W.H.); gaujang@mail2000.com.tw (K.-C.L.); d119103001@tmu.edu.tw (T.-Y.L.); d119102013@tmu.edu.tw (C.-H.H.); jayakumar@tmu.edu.tw (T.J.); 2 Department of Pharmacology, School of Medicine, College of Medicine, Taipei Medical University, Taipei 110, Taiwan; fird@tmu.edu.tw; 3 Department of Neurology, Chi Mei Medical Center, Tainan 710, Taiwan; 4 Central Laboratory, Shin‐Kong Wu Ho‐Su Memorial Hospital, Taipei 111, Taiwan; 5 Department of Chemistry, North Eastern Hill University, Shillong 793022, India; mvelusamy@gmail.com; 6 Department of Cardiovascular Center, Cathay General Hospital, Taipei 106, Taiwan; 7 Division of Cardiology, Department of Internal Medicine, School of Medicine, College of Medicine, Fu Jen Catholic University, New Taipei City 242, Taiwan

**Keywords:** arterial thrombosis, esculetin, experimental mice, human platelets, hydroxyl radical, signaling pathways

## Abstract

Esculetin, a bioactive 6,7-dihydroxy derivative of coumarin, possesses pharmacological activities against obesity, diabetes, renal failure, and cardiovascular disorders (CVDs). Platelet activation plays a major role in CVDs. Thus, disrupting platelet activation represents an attractive therapeutic target. We examined the effect of esculetin in human platelet activation and experimental mouse models. At 10–80 μM, esculetin inhibited collagen- and arachidonic acid-induced platelet aggregation in washed human platelets. However, it had no effects on other agonists such as thrombin and U46619. Esculetin inhibited adenosine triphosphate release, P-selectin expression, hydroxyl radical (OH·) formation, Akt activation, and phospholipase C (PLC)γ2/protein kinase C (PKC) phosphorylation, but did not diminish mitogen-activated protein kinase phosphorylation in collagen-activated human platelets. Platelet function analysis indicated that esculetin substantially prolonged the closure time of whole blood. In experimental mice, esculetin significantly increased the occlusion time in thrombotic platelet plug formation and reduced mortality associated with acute pulmonary thromboembolism. However, it did not prolong the bleeding time. This study demonstrates that esculetin inhibits human platelet activation via hindering the PLCγ2–PKC cascade, hydroxyl radical formation, Akt activation, and ultimately suppressing platelet activation. Therefore, esculetin may act as an essential therapeutic agent for preventing thromboembolic diseases.

## 1. Introduction

Arterial thrombosis is involved in a wide variety of coronary heart diseases, cerebrovascular diseases, and rheumatic heart diseases as well as in myocardial infarction and other conditions. Platelet adherence and aggregation is thought to initiate thrombosis. Antiplatelet agents are drugs that inhibit platelet hyperaggregability. Their main therapeutic use is to prevent vascular thromboembolic events [1]. Cyclooxygenase inhibitors [2], adenosine diphosphate (ADP) receptor antagonists [3], and glycoprotein (GP) IIb/IIIa receptor antagonists [4] are the most commonly prescribed antiplatelet agents. Responses to these drugs vary, and their use is associated with a risk of bleeding. Thus, researchers have focused on developing methods to increase their efficacy and safety as well as on the development of new aggregation modulators. Collagen receptors glycoprotein VI (GPVI) and integrin α2β1 induce phospholipase Cγ2 (PLCγ2) and protein kinase C (PKC) activation, facilitate platelet granule release [5], and then subsequently stimulate platelet aggregation. These routes are essential for platelet activation and thrombus formation. Since GPVI is an encouraging pharmacological target for the active and safe treatment of thrombotic diseases [6], inhibition of PLCγ2, PKC, and adenosine triphosphate (ATP) release are the major targets for the treatment of thrombotic diseases.

Dietary supplements are majorly contributing to various human diseases prevention, including cardiovascular diseases (CVDs). An epidemiological study revealed a link between healthy meal intake and a reduced risk of CVDs [7]. Several phytochemicals possess protective and curative potential for treating various diseases with minimal side effects. Among them, coumarins have caused a revolution in the research field because of their potential to prevent and treat several diseases [8]. Coumarins are related to the benzopyrone family (1,2-benzopyrones or 2H-1-benzopyran-2-ones) and possess a broad range of therapeutic activities. Among coumarins, esculetin (6,7-dihydroxy derivative of coumarin) is categorized as a simple coumarin. It is also known as cichorigenin and is found in numerous plants, such as *Artemisia capillaries* and *Cortex fraxini* [9].

Esculetin exhibits several pharmacological activities, including anti-inflammatory, anti-diabetes mellitus, and anti-carcinogenic [10]. Studies have found that esculetin has protective effects on the cardiovascular system. Oral pretreatment with esculetin exerted an anti-lipoperoxidative effect in rats with isoproterenol-induced myocardial infarction, which might be due to its free radical scavenging properties [8]. Furthermore, esculetin diminished smooth muscle cell proliferation and cell cycle in a rat model of balloon angioplasty and matrix metalloproteinase-9 expression in vascular smooth muscle cells [11,12]. These results indicate that esculetin is a promising therapeutic approach for CVDs and atherosclerosis. Natural coumarin derivatives (e.g., warfarin) have clinical use as anticoagulant agents [13]. However, it should be highlighted that anticoagulant drugs, such as warfarin, belonging to the dicoumarol group owe their effect to the inhibition of the enzyme vitamin K epoxide reductase, which catalyzes the step of reduced activity. Such a mechanism is not correlated with monocoumarins such as esculetin, which have no direct or indirect effect on coagulation [14].

Esculetin has been found to significantly inhibit platelet activation in rabbits [15] and rats [16]. However, very few studies have been published to date on the effect of esculetin on human platelets, and its systematic investigation on human platelet activation has yet to be done. Thus, here we thoroughly examined the detailed mechanisms of esculetin upon inhibiting platelet activation both ex vivo and in vivo.

## 2. Results

### 2.1. Effects of Esculetin on the Aggregation of Washed Human Platelets Stimulated by Various Agonists

**Figure 1:**
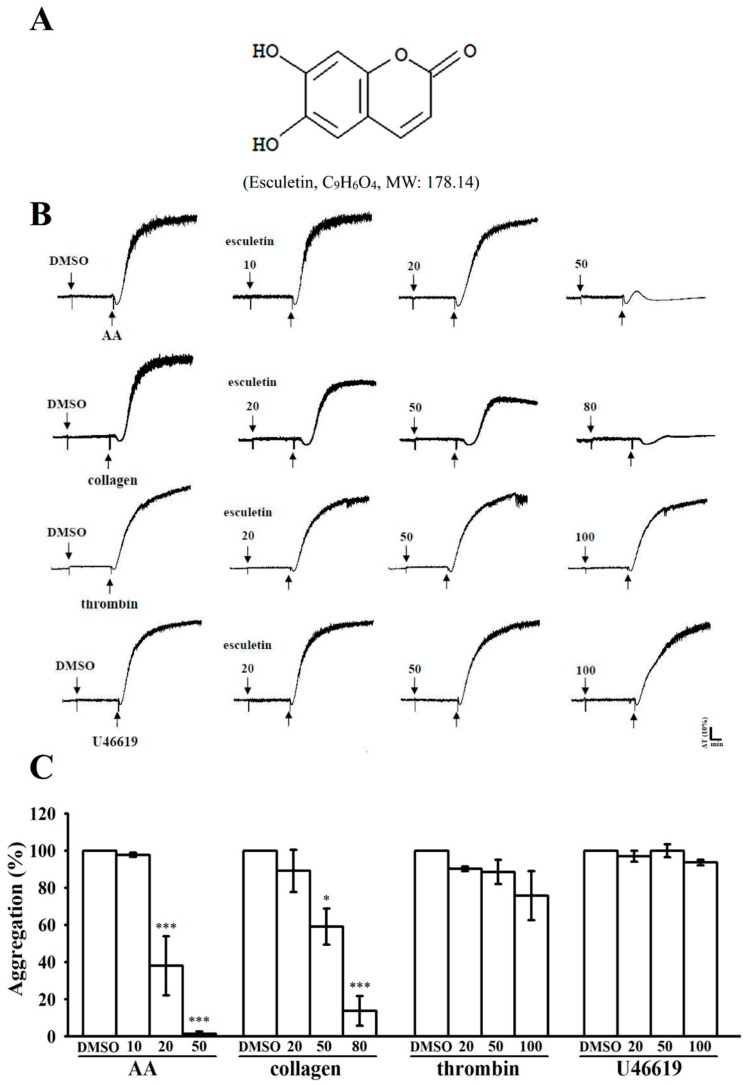
Esculetin inhibits agonists-induced platelet aggregation in washed human platelets. (**A**) Chemical structure of esculetin (C_9_H_6_O_4_). (**B**) Washed human platelets (3.6 × 10^8^ cells/mL) were preincubated with the solvent control (0.1% DMSO) or esculetin (10–100 μM) and subsequently treated with 1 μg/mL collagen, 0.01 U/mL thrombin, 1 μM U46619, and 60 μM AA to stimulate platelet aggregation. The aggregation curves in human platelets were monitored using lumi-aggregometer (Payton Associates, Scarborough, ON, Canada). ΔT/min = change in light transmission per min. (**C**) Concentration–response histograms of esculetin demonstrating its inhibitory activity for platelet aggregation (%). All data are presented as mean ± standard error of the mean (*n* = 4). * *p* < 0.05 and *** *p* < 0.001 vs. DMSO-treated group.

Esculetin (10–80 μM) (Figure 1A) exhibited strong effectiveness for inhibiting platelet aggregation stimulated by arachidonic acid (AA; 60 µM) and collagen (1 μg/mL), but not by thrombin (0.01 U/mL) or 1 µM 9,11-dideoxy-11α,9α-epoxymethanoprostaglandin (U46619), a prostaglandin endoperoxide (Figure 1B,C). Both AA- and collagen-induced platelet aggregation was completely inhibited by esculetin at 80 μM concentration. The 50% inhibitory concentration (IC_50_) of esculetin showed IC_50_ value of 50 μM against collagen-stimulated platelet aggregation. Therefore, the IC_50_ (50 µM) and maximal concentration (80 µM) of esculetin were used to examine its possible mechanisms in platelet activation stimulated by collagen, which acts as a crucial endogenous platelet activator and has a critical role in arterial thrombosis [17]. A 0.1% dimethyl sulfoxide (DMSO), used as the solvent control, did not alter platelet aggregation (Figure 1B).

### 2.2. Influence of Esculetin on Surface P-Selectin Expression, ATP Release Reaction, and Cytotoxicity in Washed Human Platelets 

**Figure 2:**
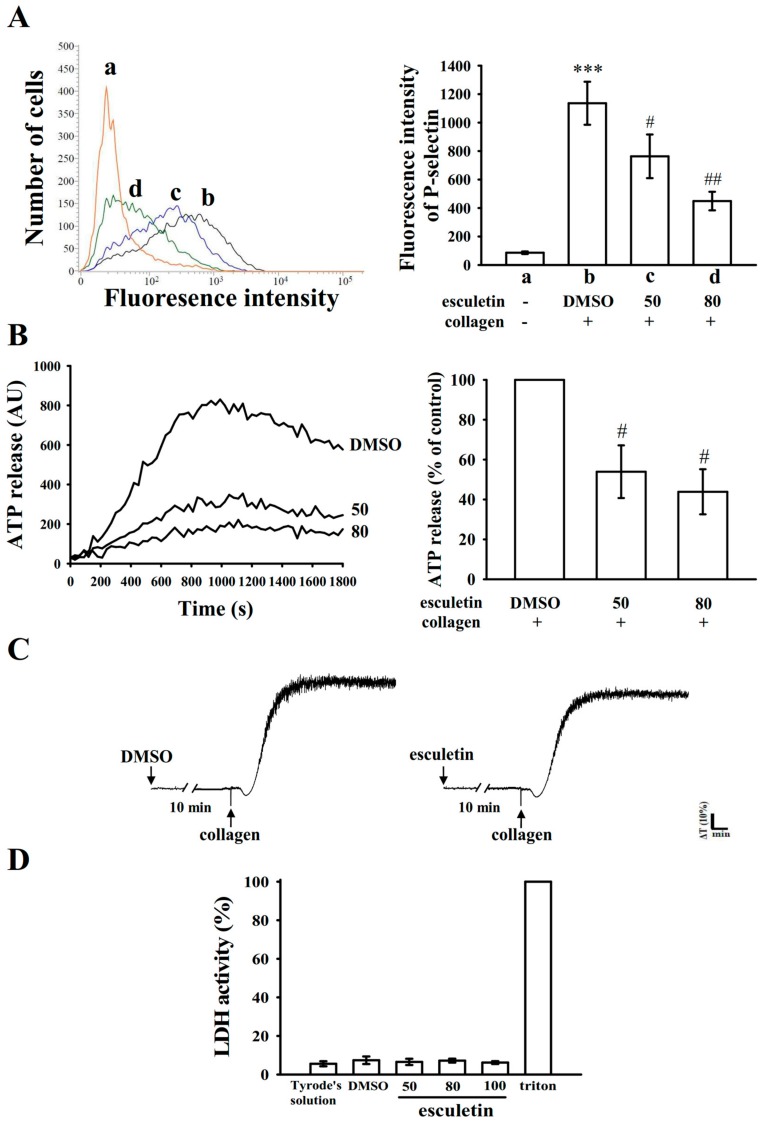
Effect of esculetin on surface P-selectin expression, ATP release, cytotoxicity, and LDH release in human platelets. Washed platelets (3.6 × 10^8^ cells/mL) were preincubated with 0.1% DMSO, esculetin (50 and 80 µM), or FITC–P-selectin (2 µg/mL); collagen (1 μg/mL) was then added to trigger either (**A**) surface P-selectin expression or (**B**) ATP release (AU: arbitrary unit). The right panel of each figure shows the respective statistical data in (**A**,**B**). For other experiments, (**C**) washed platelets were pretreated with 0.1% DMSO or esculetin (100 μM) for 10 min and subsequently double washed by Tyrode’s solution; collagen (1 μg/mL) was added to activate platelet aggregation. ΔT/min = change in light transmission per min. (**D**) Washed platelets were preincubated with 0.1% DMSO or esculetin (50, 80, and 100 µM) for 20 min, and a 10-µL suspension of the supernatant was deposited on a Fuji Dri-Chem slide (LDH-PIII). Data are presented as mean ± standard error of the mean (*n* = 4). Profiles in (**C**) are representative of four independent experiments. *** *p* < 0.001 vs. resting control; ^#^ *p* < 0.05 and ^##^ *p* < 0.01 vs. DMSO-treated group.

The release of granular substances (e.g., P-selectin expression from α-granules and adenosine triphosphate (ATP) release from dense granules), activate considerable platelet activation. Platelet activation is associated with increasing P-selectin expression, which is a critical biomarker of platelet activation. P-selectin is placed on the inner walls of α-granules, in normal conditions, whereas when platelet activate they expose the inner walls of the granules to the outer parts of the cells [16]. In this study, treatment with esculetin markedly suppressed surface P-selectin expression stimulated by collagen (resting control, 86.3 ± 9.0; collagen-activated platelets, 1136.0 ± 151.1; 50 μM esculetin, 763.3 ± 153.4; 80 μM esculetin, 449.0 ± 65.3; *n* = 4; Figure 2A). The right-hand panels of Figure 2A present the corresponding statistical data. Furthermore, esculetin (50 and 80 µM) concentration dependently diminished the ATP release reaction stimulated by collagen (1 μg/mL) (Figure 2B). Esculetin (100 μM) pretreatment for 10 min, following double wash by Tyrode’s solution did not significantly change the aggregation curves, as it is identical from those of platelets preincubated 0.1% DMSO (Figure 2C). Based on this observation, the toxicity of esculetin in platelets was preliminarily ruled out. Moreover, the lactate dehydrogenase (LDH) study revealed that platelets incubated with esculetin (50, 80, and 100 μM) for 20 min did not exhibit significantly increased LDH activity, and thus esculetin did not exert cytotoxic effects on platelets (Figure 2D). These findings demonstrated that esculetin does not affect platelet permeability or induce platelet cytolysis.

### 2.3. Regulatory Roles of Esculetin in Akt Activation and the PLCγ2–PKC Cascade

**Figure 3:**
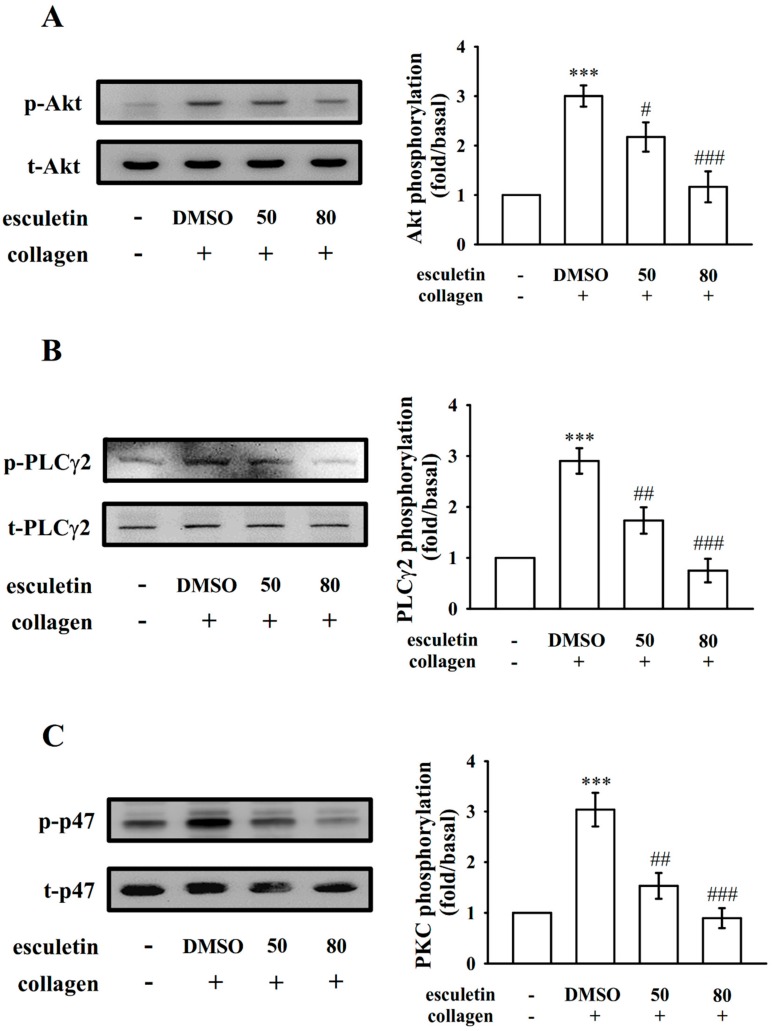
Effects of esculetin on Akt, PLCγ2, and PKC activation in human platelets. Washed platelets (1.2 × 10^9^ cells/mL) were preincubated with 0.1% DMSO or esculetin (50 and 80 µM) and subsequently added collagen (1 μg/mL) to induce (**A**) Akt phosphorylation, (**B**) PLCγ2, and (**C**) PKC activation (p47 [pleckstrin] phosphorylation). Platelets were prepared and their suspension were analyzed to determine levels of protein phosphorylation. Data are presented as mean ± standard error of the mean (*n* = 4). *** *p* < 0.001 vs. the resting control; ^#^ *p* < 0.05, ^##^ *p* < 0.01, and ^###^ *p* < 0.001 vs. DMSO-treated group.

Akt, termed as protein kinase B, is involved in the phosphatidylinositol 3-kinase [PI3K]-Akt pathway [18], and it regulates cell proliferation, apoptosis, migration as well as platelet activation. In the present study, esculetin (50 and 80 µM) evidently reduced collagen-induced Akt phosphorylation (Figure 3A). PLC hydrolyzes phosphatidylinositol 4,5-bisphosphate to generate the secondary messengers inositol 1,4,5-trisphosphate (IP_3_) and diacylglycerol (DAG). DAG activates protein kinase C (PKC), giving almost a 47-kDa protein that is predominantly phosphorylated (p47 or pleckstrin) and causes ATP release [19]. Here, as previously stated, esculetin markedly reduced ATP release (Figure 2B). We further inspected the effect of esculetin on the phosphorylation of the PLCγ2–PKC signaling cascade and found that this compound reduced phosphorylation of both PLCγ2 and p47 in collagen-activated platelets.

### 2.4. Effects of Esculetin on MAPK Activation

**Figure 4:**
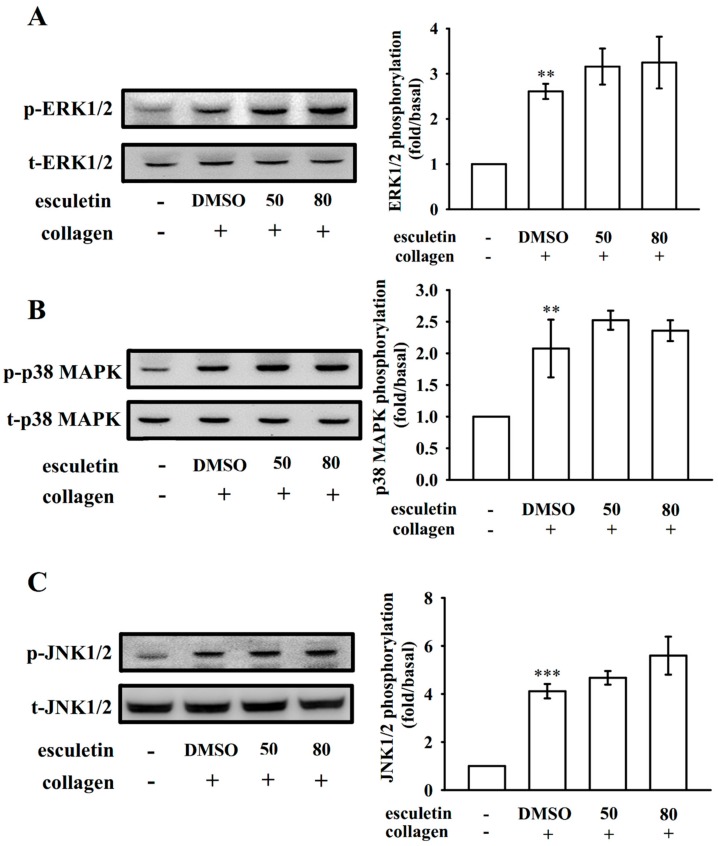
Effects of esculetin on ERK1/2, p38 MAPK, and JNK1/2 phosphorylation in collagen-activated human platelets. Washed platelets (1.2 × 10^9^ cells/mL) were preincubated with 50 or 80 μM esculetin or 0.1% DMSO and subsequently treated with 1 μg/mL collagen to induce platelet activation. Platelets were collected and subcellular extracts were analyzed to determine the phosphorylation of (**A**) ERK1/2 (**B**) p38 MAPK, and (**C**) JNK1/2. Data are expressed as mean ± standard error of the mean (*n =* 4). ** *p* < 0.01 and *** *p* < 0.001 vs. the resting control.

The MAPK signaling pathway, which includes ERK1/2, JNK1/2, and p38 MAPK, regulates inflammation, cell proliferation, apoptosis, and platelet activation [20]. Notably, esculetin (50 and 80 µM) did not diminish ERK1/2 (Figure 4A) or p38 MAPK (Figure 4B) or even JNK1/2 (Figure 4C) activation stimulated by collagen, indicating that the MAPK signaling pathway may not be involved in esculetin-mediated antiplatelet activation.

### 2.5. Esculetin Scavenges OH

**Figure 5:**
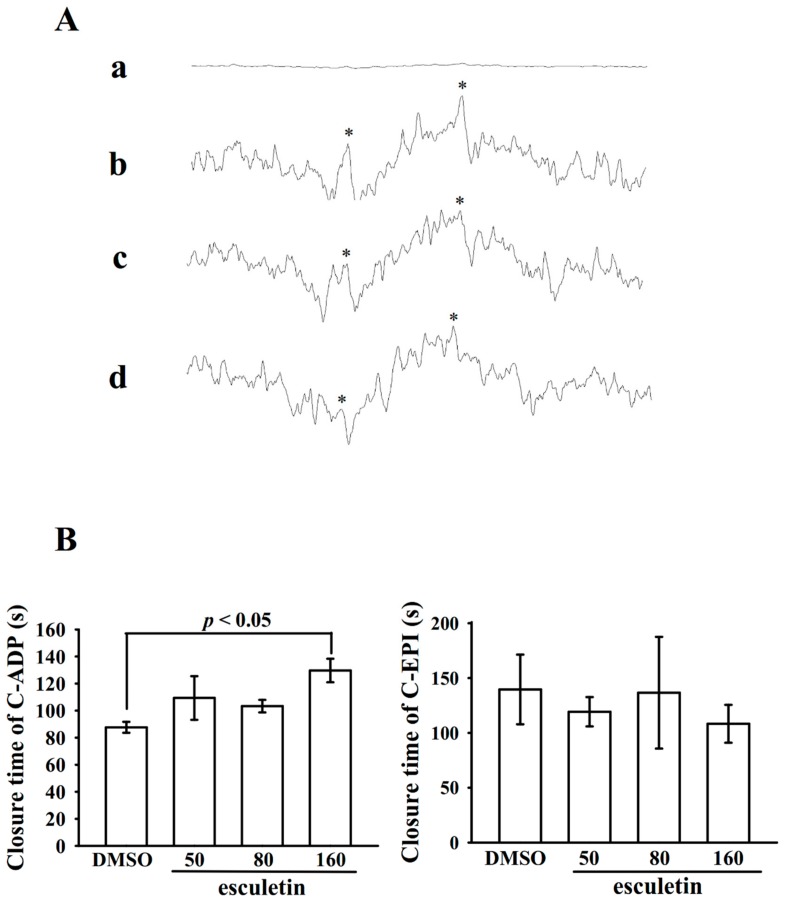
Esculetin inhibits OH· formation in platelet suspensions and the closure time in human whole blood, which are estimated by the respective electron spin resonance (ESR) and PFA-100 analyses. (**A**) Washed human platelets was incubated with (a) Tyrode’s solution only (resting control); or with (b) 0.1% DMSO, esculetin at (c) 50 µM or (d) 80 µM, collagen (1 µg/mL) was then added for the ESR experiments, as described in the Section 4 (*n* = 4). Asterisk (*) indicates OH· formation. (**B**) Platelet plug formation induced by shear stress in whole blood was determined via recording the closure time in collagen-ADP (C-ADP)- and collagen-EPI (C-EPI)-coated membranes, as described in the Section 4. Data are presented as mean ± standard error of the mean (*n* = 6); *p* < 0.05 vs. DMSO-treated group.

OH· radical formation was noted during pretreatment with 0.1% DMSO, followed by the addition of collagen to platelet suspensions (Figure 5A(b)), compared with the resting control (Figure 5A(a)). This was evidenced by the ESR signals (arrows). The collagen-induced OH· signals in platelet suspensions were significantly reduced by esculetin (50 and 80 μM, Figure 5A(c,d)).

### 2.6. Effects of Esculetin on Antithrombotic Activity Ex Vivo and In Vivo

**Figure 6:**
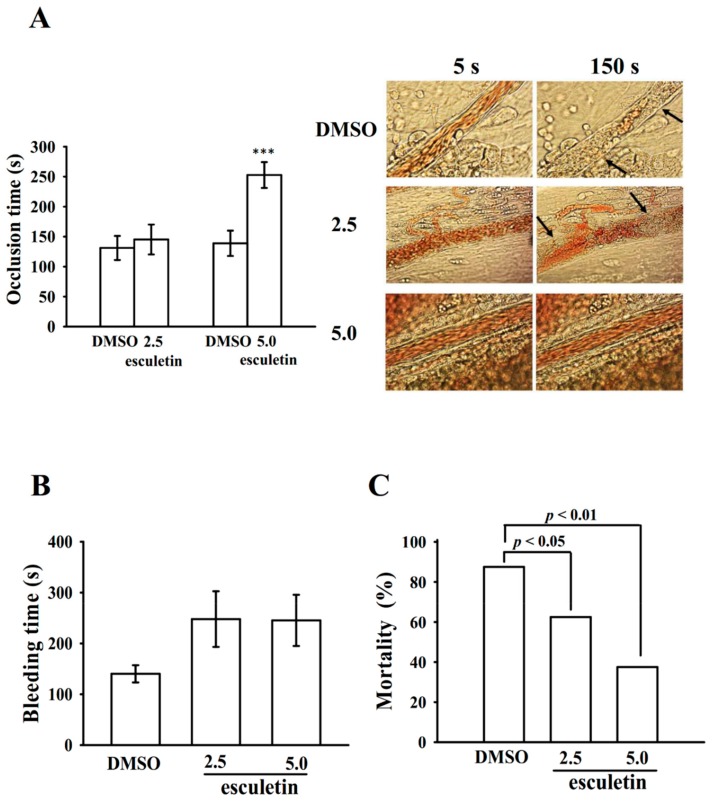
The in vivo activities of esculetin in thrombotic platelet plug formation in mesenteric venules, tail bleeding time, and acute pulmonary thromboembolism, in experimental mice. (**A**) Mice were intravenously administered 0.1% DMSO or esculetin (2.5 or 5.0 μg/kg) (all in 50 μL), and the mesenteric venules were irradiated to induce microthrombus formation (occlusion time), as described in the Section 4. Microscopic images (×400 magnification) of DMSO-treated controls and esculetin (2.5 and 5.0 μg/kg)-treated groups were recorded at 5 and 150 s after irradiation, and the platelet plug formation represents by arrows. (**B**) The bleeding time was measured through the transection of mice tails after 30 min of intraperitoneal administration of either 0.1% DMSO or 2.5/5.0 μg/kg esculetin. (**C**) For the study of acute pulmonary thrombosis, 0.1% DMSO or esculetin at various doses (2.5 and 5.0 μg/kg) was administered intraperitoneally to mice, and ADP (0.7 mg/g) was then injected through the tail veins. Data are presented as mean ± standard error of the mean (*n* = 8); *** *p* < 0.001 vs. 0.1% DMSO-treated group.

The platelet plug formation induced by shear stress was examined ex vivo in human whole blood. The used PFA-100 instrument mimics the in vivo conditions of blood vessel injury, in which platelets were exposed to a high shear rate. The CTs of C-ADP and C-EPI in solvent control (0.1% DMSO) samples were 87.7 ± 4.1 s and 139.7 ± 31.8 s (Figure 5B), respectively. After treatment with 50, 80, and 160 μM esculetin, the CTs of C-ADP were 109.3 ± 16.1 s (*p* > 0.05; *n* = 6), 103.3 ± 4.6 s (*p* > 0.05; *n* = 6), and 117.0 ± 17.3 s (*p* < 0.05; *n* = 6), respectively (Figure 5B). However, after treatment with various concentrations of esculetin (50–160 μM), the CTs of C-EPI were not significantly prolonged compared with those for the solvent control (*p* > 0.05; *n* = 6) (Figure 5B). Furthermore, we investigated the effect of esculetin on intraluminal thrombus formation in vivo. The occlusion time in microvessels pretreated with 15 µg/kg fluorescein sodium was approximately 100 s. When esculetin was administered after pretreatment with fluorescein sodium, the occlusion time for 5.0 µg/kg, but not 2.5 µg/kg, esculetin treatment was significantly prolonged compared with that for the 0.1% DMSO control (0.1% DMSO-treated, 131.1 ± 20.1 s; 2.5 µg/kg esculetin, 145.2 ± 24.9 s, *p* > 0.05, *n* = 8; 0.1% DMSO-treated, 138.9 ± 21.2 s; 5.0 µg/kg esculetin, 252.8 ± 21.5 s, *p* < 0.001, *n* = 8; Figure 6A). The thrombotic platelet plug was seen at 150 s irradiated mesenteric microvessels, but not at 5 s both 0.1% DMSO- and 2.5 µg/kg esculetin-treated mice (Figure 6A, arrows). When 5.0 µg/kg esculetin was administered, platelet plug formation was not observed 150 s after irradiation (Figure 6A). In addition, we investigated tail transection in mice 30 min after esculetin was administered intraperitoneally; the bleeding times were 140.3 ± 17.0 s (0.1% DMSO-treated group; *n* = 8), 248.0 ± 54.8 s (2.5 µg/kg esculetin-treated group; *n* = 8), and 245.4 ± 50.4 s (5.0 µg/kg esculetin-treated group; *n* = 8) (Figure 6B). To detect any rebleeding, mice were watched individually for 15 min, even bleeding was stopped. The results suggested that esculetin slightly prolonged the bleeding time, but this finding did not reach statistical significance, showing that esculetin reduced platelet plug formation in vivo, without a significant change on prolonging the bleeding time. This result is corroborated to the finding of Kwon et al., 2015, where they have propsoed that oral administration of 50 mg/kg chloroform fraction of *Euphorbia maculate*, an annual plant, to rats significantly reduced ADP-induced platelet aggregation without increasing the tail bleeding time [21]. Moreover, as shown in Figure 6C, the therapeutic effects of esculetin on preventing acute pulmonary embolism death in mice was investigated. The results indicated that treatment with 2.5 and 5.0 µg/kg esculetin significantly dropped the mortality rate in mice challenged with ADP (0.7 mg/g) compared with the 0.1% DMSO-treated controls, with percentage of death being 87.5% (7 dead, *n* = 8), 62.5% (5 dead, *n* = 8), and 37.5% (3 dead, *n* = 8), respectively.

## 3. Discussion

Our results indicate that esculetin exhibits antiplatelet activity for human platelets and effectively inhibits arterial thrombogenesis in vivo. A previous study examined the pharmacokinetics of esculetin by evaluating its absorption, distribution, metabolism, and excretion in rat plasma and tissues [22]. They found that the maximum plasma level of esculetin was higher at 5 min post oral administration, and its half-life in plasma was reported to be 45 min [22]. Therefore, esculetin’s pharmacokinetic profile suggests that its absorption and elisecation from plasma is fast, and that it may quickly distributed and eliminated from tissues. Normal consumption of esculetin from natural sources would not be adequate to obtain the required plasma concentrations that can inhibit in vivo platelet activation. As it is stated that long-term intake of sufficient natural food products or nutritional supplements is ideal to inhibit atherothrombotic events, esculetin may serve as an innovative antithrombotic agent in humans.

Esculetin effectively inhibited platelet aggregation induced by collagen and AA, but not that stimulated by thrombin or U46619. Esculetin has been reported to abolish rat platelet aggregation by inhibiting lipoxygenase (LOX) [16]. Human LOX enzymes include 5-LOX, 12-LOX (with platelet- and leukocyte-type forms), and 15-LOX [23]. AA is released from cell membranes by phospholipases such as phospholipase A in response to various cytokines, peptides, and growth factors that become active under inflammatory conditions [23]. Platelet 12-LOX is involved in the oxidative metabolism of AA and produces leukotrienes, 12-hydroperoxyeicosatetraenoic acid (12-HPETE), and 12-hydroxyeicosatetraenoic acid (12-HETE), which are involved in platelet activation [23]. This may explain why esculetin exhibits higher efficacy for inhibiting AA-triggered platelet aggregation than for inhibiting platelet aggregation triggered by other agonists in this study. Furthermore, the activation of platelets by agonists such as collagen substantially alters phospholipase activation. The activation of PLC results in IP_3_ and DAG production, which activates PKC, thereby inducing the phosphorylation of the p47 protein [24]. PKC activation facilitates the occurrence of particular responses to specific activating signals in distinct cellular compartments [25]. PLC enzymes can be classified into the following six families: PLCβ, PLCγ, PLCδ, PLCε, PLCζ, and PLCη [26]. The PLCγ family comprises isozymes 1 and 2. PLCγ2 is involved in collagen-dependent signaling in platelets [26]. The current study suggested that PLCγ2-PKC signaling involve esculetin-mediated inhibition of platelet activation.

MAPK subfamilies exist in mammalian cells are ERK1/2 (also called p44/42MAPK), JNK1/2, and p38 MAPK. All of which are triggered by specific MAPK kinases (MEKs); specifically, MEK1/2, MEK3/6, and MEK4/7 activate ERK1/2, p38 MAPK, and JNK1/2, respectively [27]. These three major MAPKs have been identified in platelets [28]. Various agonists, including von Willebrand factor (vWF) and thrombin induce cytosolic phospholipase A_2_ (cPLA_2_), a substrate of p38 MAPK [29]. ERK activation induces platelet aggregation, in which ATP release play role via inducing P_2_X_1_-mediated Ca^2+^ influx, thereby increasing the phosphorylation of myosin light chain kinase [30]. As JNK1 is the newly identified MAPK in platelets, its activation is poorly established in platelet aggregation. JNK1 is activated by several agonists, including thrombin, vWF, collagen, and ADP [31]. However, the present study found that esculetin-mediated inhibition of collagen-triggered platelet activation did not involve MAPK activation. This finding also clarifies that esculetin shows relatively weak activity for inhibiting platelet aggregation stimulated by either thrombin or U46619. Furthermore, Akt is a downstream effector of PI3K [32], and Akt-knockout mice exhibited defects in agonist-induced platelet activation [33,34]. Our previous study showed that the upstream regulators of PKC, such as PI3K/Akt was activated in induced platelets [35]. Therefore, protein kinases contributing to Akt activation, particularly PI3Kβ, may be attractive targets for the development of antithrombotic therapeutics.

Reactive oxygen species, such as hydrogen peroxide and hydroxyl radicals, may increase platelet activation during in vivo thrombus formation. In addition, hydrogen peroxide formation in platelets is converted into hydroxyl radicals. During the initial phase of platelet activation, free radicals act as secondary signals, and PKC is involved in receptor-mediated free radical production in platelets [36]. Esculetin possesses a structural coumarin moiety and two hydroxyl substitutions, and it has potent antioxidant activity; evidence has been presented for its ability to quench free radical such as DPPH radical and hydroxyl radicals [9]. The results of our ESR analysis prove that esculetin scavenges OH· in human platelets. Thus, a part of the reason for esculetin-mediated inhibition of thrombogenesis in vivo may be its free radical scavenging activity. Exposure of subendothelial collagen triggers platelet adhesion and aggregation at the site of vascular endothelial cell injury, followed by arterial thrombus formation. The time required for platelet aggregation to occlude an aperture in a collagen-coated membrane can be recorded by PFA-100 instrument. Platelet adhesion to collagen depends on flow conditions. In this study, platelets depleted of activity were unable to adhere to collagen under flow conditions.

In our in vivo study on thrombosis, mesenteric venules were continuously irradiated with fluorescein sodium throughout the experimental period, leading to strong damage to endothelial cells. In this study, 5.0 μg/kg esculetin pointedly prolonged occlusion times, and this effect may be due to the inhibition of platelet activation. In addition, the tail transection mouse model was used to examine the antithrombotic effects of esculetin via measuring the mice tail bleeding time. Despite aspirin being used as an effective antiplatelet drug to treat cardiovascular and cerebrovascular diseases, it has limitation due to the unwanted prolongation of bleeding time. Here, we found that the bleeding times of esculetin-treated mice did not increase significantly, as it was slightly increased than that of the solvent control (0.1% DMSO)-treated mice. It indicates that esculetin possesses antiplatelet activity with low bleeding risk. Furthermore, as platelet aggregation is closely associated in experimental thrombosis, in this study, as expected, esculetin successfully prevented ADP-induced thromboembolic death. By contrast, this study noticed that heparin (1.5 U/g) could not reduce the ADP-induced mortality in mice (data not shown). These data are consistent with the fact that platelet aggregation is a crucial factor causing thromboembolism than that in fibrin formation. The findings of this study may reveal that esculetin could act as a novel antiplatelet agent, and hence propose that it can be used for the prophylactic applications.

## 4. Materials and Methods

### 4.1. Materials

Esculetin, collagen (type I), luciferin-luciferase, arachidonic acid (AA), 9,11-dideoxy-11α,9α-epoxymethanoprostaglandin (U46619), 5,5-dimethyl-1 pyrroline N-oxide (DMPO), heparin, prostaglandin E_1_ (PGE_1_), and thrombin were purchased from Sigma (St. Louis, MO, United States). Anti-phospho-p38 mitogen-activated protein kinase (MAPK) Ser^182^ monoclonal antibody (mAb) was purchased from Santa Cruz (Santa Cruz, CA, United States). Anti-p38 MAPK and anti-phospho-JNK (Thr^183^/Tyr^185^) mAbs and anti-phospholipase C (PLC)γ2, anti-phospho (Tyr^759^) PLCγ2, and anti-phospho-p44/p42 extracellular signal-regulated kinase (ERK) (Thr^202^/Tyr^204^) polyclonal antibodies (pAbs) were purchased from Cell Signaling (Beverly, MA, United States). Anti-phospho-Akt (Ser^473^) and anti-Akt mAbs were purchased from Biovision (Mountain View, CA, United States). Anti-α-tubulin mAb was purchased from NeoMarkers (Fremont, CA, United States). Hybond-P polyvinylidene difluoride (PVDF) membranes, an enhanced chemiluminescence (ECL) Western blotting detection reagent, horseradish peroxidase (HRP)-conjugated donkey anti-rabbit immunoglobulin G (IgG), and sheep anti-mouse IgG were purchased from Amersham (Buckinghamshire, United Kingdom). Dade Behring platelet function analysis (PFA) collagen-epinephrine (C-EPI) and collagen-ADP (C-ADP) test cartridges were obtained from Siemens Healthcare (Erlangen, Germany). Esculetin was dissolved in 0.1% dimethyl sulfoxide (DMSO) and stored at 4 °C.

### 4.2. Platelet Aggregation

The Institutional Review Board of Taipei Medical University was approved this study (Taipei, Taiwan; approval no. TMU-JIRB-N201612050) and further conformed to the directives of the Helsinki Declaration. Human volunteers who had involved this study provided informed consent, and platelets suspensions were prepared as described previously [37]. Blood was collected from healthy human donors (age: 20–30 years; no any history of abnormal bleeding, diabetes mellitus, and arterial or venous thrombotic disorders) who did not take any medication during the 2 weeks prior to collection. This blood was mixed with acid-citrate-dextrose solution. After centrifugation, the supernatant (platelet-rich plasma) was subjected to 0.5 μM PGE_1_ and 6.4 IU/mL heparin for washing. Pure washed platelets were suspended in Tyrode’s solution containing 3.5 mg/mL bovine serum albumin (BSA). The final concentration of Ca^2+^ in Tyrode’s solution was 1 mM.

Platelet aggregation was monitored using lumi-aggregometer (Payton Associates, Scarborough, ON, Canada) as previously described [37]. The suspensions of platelet (3.6 × 10^8^ cells/mL) were preincubated with esculetin (10–80 μM) or an isovolumetric solvent control (final concentration, 0.1% DMSO) for 3 min before agonists were added. The reaction proceeded for 6 min, and the level of aggregation was calculated in light transmission units. The adenosine triphosphate (ATP) release was measured by adding 20 μL of the luciferin-luciferase mixture, 1 min before the addition of agonists. The amount of ATP was determined with that released by the control.

### 4.3. Lactate Dehydrogenase (LDH) Activity

Washed platelets (3.6 × 10^8^ cells/mL) were preincubated with esculetin (50–100 μM) or the solvent control (0.1% DMSO) for 20 min at 37 °C. An aliquot of the supernatant (10 μL) was deposited on a Fuji Dri-Chem slide (LDH-PIII; Fuji, Tokyo, Japan), and absorbance was read at the wavelength of 540 nm on an ultraviolet-visible spectrophotometer (UV-160; Shimadzu, Kyoto, Japan). A maximal value of LDH was observed in sonicated platelets.

### 4.4. Immunoblotting

Washed platelets (1.2 × 10^9^ cells/mL) were preincubated with 50 or 80 μM esculetin or 0.1% DMSO for 3 min, and collagen (1 μg/mL) was consequently added to induce platelet activation. After the reaction stopped, platelets were immediately resuspended in 200 μL of lysis buffer. Total 80 μg of protein was separated on 12% acrylamide gel through SDS-PAGE, and proteins were electrotransferred to the PVDF membranes using a semidry transfer unit (Bio-Rad, Hercules, CA, United States). Membranes were blocked with TBST (10 mM Tris-base, 100 mM NaCl, and 0.01% Tween 20) containing 5% BSA for 1 h and probed with various specific primary antibodies. The membranes were incubated with HRP-linked anti-mouse IgG or anti-rabbit IgG (diluted 1:3000 in TBST) for 1 h. Immunoreactive bands were detected using an ECL system. Relative protein analysis was performed by using video densitometer and the Bio-Profil Biolight software package (Version V2000.01; Vilber Lourmat, Marne-la-Vallée, France).

### 4.5. Detection of Hydroxyl Radical Formation 

Electron spin resonance (ESR) spectrometry was achieved using a Bruker EMX ESR spectrometer (Bruker, Billerica, MA, United States), as described previously [38]. Platelet suspensions (3.6 × 10^8^ cells/mL) were preincubated with 50 or 80 μM esculetin or 0.1% DMSO for 3 min before 1 μg/mL collagen was added. The suspensions were incubated for 5 min, and 100 μM DMPO was added before ESR analysis was performed. The ESR spectrometer was operated at a power of 20 mW, frequency of 9.78 GHz, scan range of 100 G, and receiver gain of 5 × 10^4^. The rate of free radical scavenging activity was calculated using the following equation: inhibition rate = 1 − [signal height (esculetin)/signal height (solvent control)] [38].

### 4.6. Measurement of Closure Time of Human Whole Blood Using a PFA-100™ Platelet Function Analyzer

Platelet functions were analyzed using a platelet function analyzer (PFA-100 system; Dade Behring, Marburg, Germany) according to the method described in a previous study [39]. First, 0.8 mL of human whole blood was treated with esculetin (50–160 μM) or the solvent control (0.1% DMSO) for 2 min, poured onto cartridges containing C-ADP– or C-EPI–coated membranes, and subjected to a high shear rate of 5000–6000 /s. A platelet plug formed, which then slowly occluded the aperture; subsequently, blood flow gradually decreased and finally stopped. The time required to obtain full occlusion of the aperture by the platelet clot was defined as the “closure time” and was recorded in the collagen membrane [39].

### 4.7. Fluorescein-Induced Platelet Thrombi in Mesenteric Microvessels of Mice

Our protocols conformed to the Guide for the Care and Use of Laboratory Animals (8th edition, 2011) and were approved by the Institutional Animal Care and Use Committee of Taipei Medical University (LAC-2016-0276, 2017.08.01). Before animals underwent the experimental procedures, they were all clinically normal and free from infection or inflammation. The formation of thrombus was measured as previously described [40]. Male ICR mice (6 weeks old) were anesthetized, and their external jugular vein was cannulated with a PE-10 tube to administer dye and drugs intravenously. For microthrombus formation, venules (30–40 μm) were selected for irradiation at wavelengths below 520 nm. Various doses of esculetin (2.5 and 5.0 μg/kg) or 0.1% DMSO (all in 50 μL) were administered 1 min after the administration of sodium fluorescein (15 μg/kg), and the time required for the microvessels to be occluded as a result of thrombus formation (occlusion time) was recorded.

### 4.8. Bleeding Time in Mice Tail Veins

The measurement of bleeding time was performed by transecting the tails of the male ICR mice. In brief, mice were anesthetized and administered 2.5 and 5.0 μg/kg esculetin or 0.1% DMSO (all in 50 μL) intraperitoneally for 30 min. To measure the tail bleeding time, the tails were cut 3 mm from the tip and directly placed in a tube containing with normal saline at 37 °C. Bleeding time was recorded till the blood leakage was completely stopped.

### 4.9. ADP-Induced Acute Pulmonary Thromboembolism in Mice

Acute pulmonary thromboembolism was induced according to a previously described method [41]. Various doses of esculetin (2.5 and 5.0 μg/kg) or 0.1% DMSO (all in 50 μL) were injected intraperitoneally to mice. After 5 min, ADP (0.7 mg/g) was injected into the tail vein. The mortality rate of mice was observed in all groups within 10 min after the injection.

### 4.10. Statistical Analysis

The experimental results are expressed as the mean ± standard error of the mean. Values of *n* refer to the number of experiments, and each experiment was conducted using different blood donors. The significant value among the experimental groups in mice was analyzed by using unpaired Student’s *t* test. Variations between the experimental setup were calculated using One-way analysis of variance (ANOVA). If this analysis showed significant differences between the group, they were compared using the Student–Newman–Keuls method; *p* < 0.05 indicated statistical significance. Statistical analyses were performed using SAS Version 9.2 (SAS Inc., Cary, NC, United States).

## 5. Conclusions

Esculetin inhibits platelet activation by inhibiting the PLCγ2–PKC–Akt cascade and hydroxyl radical formation, ultimately inhibiting platelet aggregation. Our findings suggest that esculetin might be therapeutically considered as an innovative agent for treating thromboembolic disorders.
